# Tubulocystic Carcinoma of the Kidney: A Case Report of Natural History and Long-Term Follow-Up

**DOI:** 10.1100/tsw.2010.56

**Published:** 2010-04-01

**Authors:** Kelvin A. Moses, John J. DeCaro, Adeboye O. Osunkoya, Muta M. Issa

**Affiliations:** ^1^ Department of Urology, Emory University School of Medicine, Atlanta, USA; ^2^ Department of Pathology, Emory University School of Medicine, Atlanta, USA; ^3^ Department of Urology, Atlanta VA Medical Center, Decatur, GA, USA

**Keywords:** tubulocystic carcinoma, renal cell carcinoma, partial nephrectomy, active surveillance

## Abstract

Tubulocystic carcinoma (TC) is a rare primary renal tumor that has been recently described in the pathology literature. Formerly termed low-grade collecting duct carcinoma, further molecular analysis has shown TC to be a distinct entity that is separate from the more aggressive collecting duct carcinoma. Previous series have described the microscopic and immunohistochemical features of this tumor. We describe the natural history of this tumor in a patient who was followed with active surveillance for several years and then underwent partial nephrectomy. Long-term follow-up has shown no evidence of disease. A review of the pertinent literature is performed.

## INTRODUCTION

Collecting duct carcinoma (CDC) is an uncommon variant of renal cell carcinoma (RCC) that has been proven to be highly aggressive and associated with poor prognosis[1]. Beginning in the mid-90s, there were reports of a potential low-grade variant of CDC that had a distinctively benign course[2]. Originally termed low-grade collecting duct carcinoma, subsequent studies have described this as a separate entity known as tubulocystic carcinoma (TC)[3,4,5]. Of the 55 previous cases described, there is a generally benign behavior of the disease, although there are some reports of patients with metastatic disease requiring further treatment[5,6,7]. The vast majority of reports of TC have been incidental findings on autopsy, or of patients who had radical or partial nephrectomy for concern of malignancy. To our knowledge, there is no previous description in the urologic literature of a case that was followed in an expectant manner, with subsequent surgery and long-term follow-up. Herein we describe a patient who underwent active surveillance with serial imaging for a small tumor, who had partial nephrectomy in conjunction with surgery for a separate disease process. This case demonstrates the nonaggressive nature of such tumors, which allows for extended active surveillance without compromising prognosis.

## CASE REPORT

A 68-year-old, Caucasian male was referred to the Urology clinic with an incidentally discovered 1.6-cm lower pole renal lesion and nearby Bosniak class 1 renal cyst identified by CT scan in 2002 (Fig. 1a). His medical history was significant for squamous cell carcinoma of the larynx (treated with external beam radiation therapy and chemotherapy 6 years prior), colonic polyps, coronary artery disease, peripheral vascular disease, hypertension, and hepatitis B and C. Mag-3 nuclear renal scan demonstrated 35% function on the right and 65% function on the left. His baseline creatinine was 0.8 mg/dl. Initial management of his renal lesion was conservative, including serial imaging with renal ultrasound and yearly CT scans, which were consistent with a slow-growing renal tumor (Fig. 1b). The most recent imaging prior to surgical excision showed that the mass measured 1.9 cm, showing minimal interval growth of 3 mm in 5 years. The patient eventually underwent exploratory laparotomy and right hemicolectomy for recurrent colonic polyps in 2007, at which time he had simultaneous right partial nephrectomy.

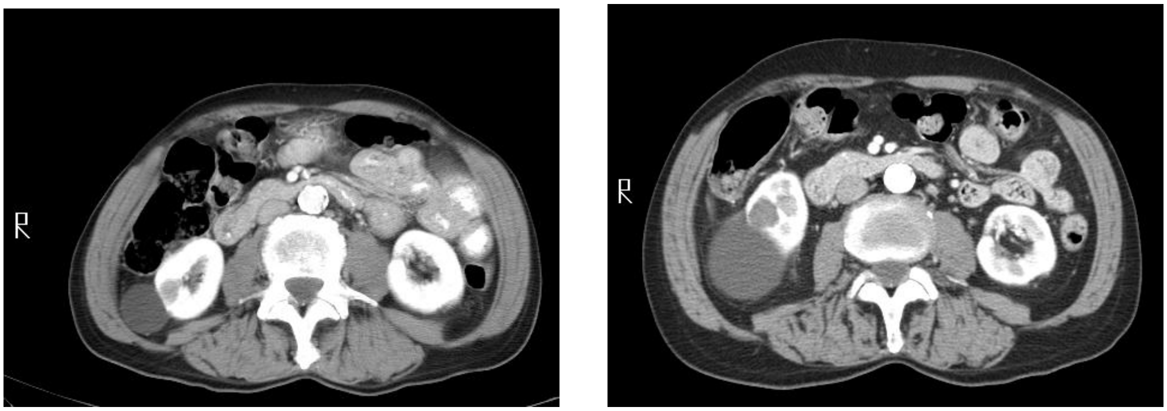

A B

**FIGURE 1.** CT imaging of right renal mass. (A) 2002, (B) 2007.

Gross examination of the renal tumor revealed a 1.7- × 1.5- × 1.3-cm cystic mass and a 4.5-cm simple cyst. Initial analysis of the cystic mass was described as low-grade CDC; however subsequent analysis of molecular and morphologic features were consistent with TC (Fig. 2a and 2b). The tumor was Fuhrman nuclear grade 2, with positive stains associated with the proximal collecting tubule (CD10, P504S/AMACAR) and distal nephron/collecting ducts (high molecular weight CK 34βE12). In addition, CK7 and EMA were also positive. Mucicarmine staining was negative. The surgical margin was negative. Thirty months postoperatively, the patient remains cancer free, with normal postoperative renal imaging and preservation of his baseline renal function. Notably, his final colonic pathology was tubular adenoma.

## DISCUSSION

TC was not formally recognized under contemporary classification systems of renal neoplasms, including the World Health Organization 2004 classification of renal tumors[5]. However, the very recent 2010 AJCC/UICC TNM, 7th edition, cancer protocol for RCC now recognizes this tumor as a distinct entity. In one of the earliest case series describing this entity, MacLennan et al. reported on 13 cases of renal tumors thought to be of collecting duct origin that had low malignant potential[2]. In their report, patients had a

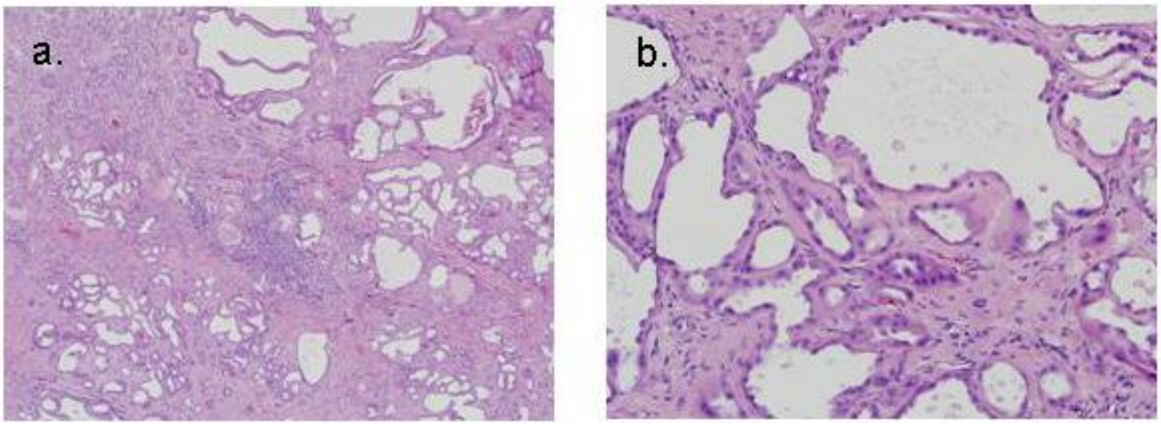

**FIGURE 2**. (a) Low magnification (2×) demonstrates numerous malignant cysts and tubules of variable sizes with occasional entrapped benign renal tubules. (b) High magnification (20×) demonstrates malignant cysts predominantly lined by a single layer of hobnail cells with mild nuclear pleomorphism and prominent nucleoli.

mean age of 61 and tumors had a mean diameter of 6 cm. Grossly, tumors were well circumscribed with minimal necrosis and low nuclear grade. Subsequently, they noted that the 13 cases were actually two distinct groups[3]. One, termed mucinous tubular and spindle cell carcinoma, is characterized by complex anastomotic tubular structures with abundant mucin. The second, TC, is characterized by immunoreactivity to UEA-1 or 34βE12, and little to no mucin production. Features of TC that are distinct from CDC include well-circumscribed tumors with low-grade appearance. Tubules and cystic structures within a fibrotic stroma are typically lined by a single layer of low cuboidal epithelial cells and eosinophilic cytoplasm, with areas of hobnail appearance. Further histopathological analysis has shown that the majority of tumors are pT1, with a small subset being pT2 or T3 (up to 10–15% each)[4,5]. TC also shares features of both proximal (CD10 and P504S) and distal (CK19) tubules[4]. Most tumors are classified as Fuhrman grade 3, however, the clinical course does not appear to correlate with nuclear grade as it does with conventional clear cell or papillary RCC. Two groups have reported metastatic disease in four patients and one local recurrence, however, there appears to be an overall benign course of the disease[4,5].

Although originally thought to be a subtype of CDC, a recent study by Osunkoya et al. has shown that TC is distinct from CDC at a molecular level[8]. TC was characterized by vimentin, p53, and α-methylacyl CoA racemase overexpression in comparison to CDC. Further work by Zhou et al. has shown that TC has a molecular profile similar, but distinct, to papillary RCC[9]. Both tumors exhibited a tendency to multicentricity and a gain of chromosomes 7 and 17, and loss of the Y chromosome. Additionally, 10 of 20 cases of TC had associated papillary RCC, with four exhibiting admixture of the two types within the same lesion.
